# Effect of Radium-223 on the Gut Microbiota of Prostate Cancer Patients: A Pilot Case Series Study

**DOI:** 10.3390/cimb44100336

**Published:** 2022-10-16

**Authors:** Ana Fernandes, Ana Oliveira, Carla Guedes, Rúben Fernandes, Raquel Soares, Pedro Barata

**Affiliations:** 1 Department Nuclear Medicine, Centro Hospitalar e Universitário de São João, E.P.E., 4200-319 Porto, Portugal; 2 Laboratory of Medical and Industrial Biotechnology, Instituto Politécnico do Porto, 4200-465 Porto, Portugal; 3 i3S—Instituto de Investigação e Inovação em Saúde, Universidade do Porto, 4200-135 Porto, Portugal; 4 Faculdade de Ciências da Saúde da Universidade Fernando Pessoa, 4200-150 Porto, Portugal; 5 Department of Biomedicine, Faculdade de Medicina da Universidade do Porto, 4200-319 Porto, Portugal; 6 Department of Pathology, Centro Hospitalar Universitário do Porto, 4099-001 Porto, Portugal

**Keywords:** prostate cancer, gut microbiota, Radium-223, ionizing radiation

## Abstract

Radium-223 (Ra-223) is a targeted nuclear medicine therapy for castration-resistant prostate cancer with bone metastases. Its major route of elimination is the intestine. There is overwhelming evidence that the gut microbiota is altered by ionizing radiation (IR) from radiotherapy treatments. Nevertheless, it is known that extrapolation of outcomes from radiotherapy to nuclear medicine is not straightforward. The purpose of this study was to prospectively determine the effect of Ra-223 on selected important bacteria from the gut microbiota. Stool samples from three prostate cancer patients and two healthy individuals were obtained, processed, and analysed. We specifically measured the relative change of the abundance of important bacteria, determined by the 2^−ΔΔC^ method. We found that Ra-223 influenced the gut microbiota composition. The most relevant changes were increases of Proteobacteria and Atopobacter; and decreases of Bacteroidetes, *Prevotella*, *Lactobacillus*, *Bifidobacterium*, *Clostridium coccoides,* and *Bacteroides fragilis*. Additionally, our experiment confirms that the composition of gut microbiota from prostate cancer patients is altered. No significant correlation was found between each subject’s gut microbiome profile and their clinical indices. Despite its limited sample, the results of this pilot study suggest that ionizing radiation from Ra-223 alters the gut microbiota composition and that the gut microbiota of prostate cancer patients has an increase of the bacteria with known prejudicial effects and a decrease of the ones with favorable effects.

## 1. Introduction

Prostate cancer is the second most frequent cancer (first in Europe), with nearly 1.4 million new diagnosis in 2020; and the fifth leading cause of cancer death among men, with 375,000 deaths worldwide in 2020 [1].

Multiple risk factors are known to precipitate prostate cancer development, progression, or treatment resistance; including age, race, family history, obesity, infection, inflammation, and other environmental factors (dietary and lifestyle) [2]. Several factors, such as bacteria, viruses, hormones, diet, and urinary reflux were suggested as potential triggers of prostate inflammation [3]. Interestingly, the current literature has demonstrated that unlike healthy prostate tissue, neoplasic prostate tissue contains bacterial DNA [4].

Previous research revealed that the microbiome from the gut, urinary tract, oral cavity, and intraprostatic may play an important role in the development of benign and malignant prostate diseases, such as prostatitis, benign prostatic hyperplasia (BPH), and prostate cancer [2].

The microbiota is thought to promote tumor oncogenesis, from initiation to progression, due to its potential to modulate inflammation, promote chronic inflammation status, and influence the genomic stability of host cells [2,3,4] through the translocation of bacteria, bacterial toxins, cytokines, hormones, or through the migration of innate and adaptive immune cells [5].

Evidence also suggests that the microbiota may be involved in the prostate tumor microenvironment and act directly or indirectly in the tumorigenesis process [3,4]. The direct mechanisms, as mentioned above, are the association of prostate cancer with chronic inflammatory urinary tract conditions, such as chronic prostatitis and BPH [4], and the influences of the gut microbiota on the metabolic processes and systemic inflammation that tend to trigger prostate tumorigenesis are considered the indirect mechanisms [4].

Prostate cancer has multiple treatment options that are chosen depending on various factors, such as the type and stage of cancer, prevalent side effects, the patient’s preferences, and overall condition. The microbiome has been suggested to modulate the efficacy of anti-cancer treatments such as chemotherapy, radiotherapy, and hormonal therapy [6,7].

Many of these anti-cancer treatment options effects are mediated by the immune system response against tumor cells, and the microbiome is known to mediate the immune system [3]. Evidence suggests that chemotherapy and radiotherapy alter the diversity and composition of the gut microbiota [8]. Studies also found that certain bacteria in the gut during androgen deprivation therapy (ADT) can synthesize androgens, thereby promoting tumor progression and therapy resistance [9].

Despite the growing number of publications regarding the relation between gut microbiota and prostate cancer therapies, to the best of our knowledge, there are no published studies regarding the influence of Radium-223 on the gut microbiota.

Radium-223 dichloride (Ra-223, Xofigo^®^) is a therapeutic targeted option for castration-resistant prostate cancer (CRPC) patients with symptomatic bone metastases and no evidence of visceral metastases [10,11]. Ra-223 emits high-energy alpha particles of short range that mainly cause double-stranded DNA breaks, resulting in a potent and highly localized cytotoxic effect in the target areas [11].

After intravenous injection, Ra-223 is rapidly cleared from the blood and selectively bound to the bone, especially in areas of high turnover, such as osteoblastic bone metastases [11]. A significant amount of activity is excreted into the intestine, constituting its major route of elimination [10]; therefore, relatively higher radiation exposure to the intestine is expected, especially in patients with constipation [10].

Although Ra-223 was reported to have a favorable safety profile [11], adverse reactions are known, the most frequent (≥10%) being diarrhea, nausea, vomiting, and thrombocytopenia [10].

There is overwhelming evidence that the gut microbiota is significantly altered by ionizing radiation, mostly from radiotherapy treatments [12,13,14,15,16,17]. Nevertheless, it is well known that extrapolation of outcomes from radiotherapy to nuclear medicine is not straightforward, not only because of differences in dose-rate effects but also because of dosimetry, linear energy transfer, duration of treatment delivery, fractionation, range, and target volume [18,19]. These differences lead to different molecular activation and cellular signaling pathways, inducing different biological responses [18,19].

Our study aimed to evaluate the effect of IR from Radium-233 on the human gut microbiota composition. Additionally, the composition of the gut microbiota from prostate cancer patients was compared with controls. In order to do this, a collection of different bacteria from human fecal samples from prostate cancer patients and healthy volunteers was obtained and studied.

## 2. Materials and Methods

### 2.1. Patient Selection

Patients with histologically confirmed progressive CPRC with two or more bone metastases detected by skeletal scintigraphy, no known visceral metastases, and adequate hematologic, liver, and renal function were included in this study.

The exclusion criteria were previous systemic nuclear medicine treatments, and treatments with antibiotics, steroids, or immunosuppressors six months prior to the study.

Seven patients scheduled to receive Ra-223 were considered. Of those seven, only four patients met the inclusion criteria. In addition, two samples from one patient were excluded from subsequent analysis due to its low quality. Three patients were included in the final analysis.

Additionally, two gender-matched healthy volunteers were recruited to provide samples as healthy controls.

### 2.2. Study Design and Sampling

Fecal samples were collected from prostate cancer patients and healthy controls. The study included two controls: self-control (same patients before starting treatment) and healthy individuals. Due to the indication of the drug, all participants in the study were males.

Two sequential stool samples were collected from each patient: before starting treatment (baseline sample, T0) and after treatment (T1). All T0 samples were collected 1 to 2 days before treatment, and all T1 samples were collected 8–10 days after the first treatment. The healthy individuals provided one sample each. A total of eight samples were collected (six from prostate cancer patients and two from healthy controls).

Each participant collected stool into a sterile plastic container, and the samples were immediately stored at the laboratory at −80° until further processing.

### 2.3. DNA Extraction and Microbiota Composition Analysis

Bacterial genomic DNA was obtained from fecal samples previously stored at −80 °C. Briefly, 40 ng of each sample was homogenized in 300 µL of ATL buffer (Qiagen™, Germantown, MD, USA). Afterward, 20 µL of proteinase K (20 mg/mL) was added and digested at 57 °C for 60 min (dry bath). The Total DNA was extracted using Lab-Aid 824 s DNA Extraction Kit (Zeesan™, Xiaquen, China). Concentration and purity (260/280 and 260/230 ratios) were determined with µDrop™ Plate (Multiskan SkyHigh Microplate Spectrophotometer, Thermo Scientific™) for each sample, and diluted to 10 ng/µL. Further amplification was performed using the NZYSpeedy qPCR Green Master Mix Kit (NZYtech–Genes & Enzymes) using the standard primer concentration according to the kit instructions (400 nM), in a qTOWER3 Real-Time PCR Thermal Cycler (Analytik Jena, Jena, Germany) under the following cycling conditions: Incubation at 95 °C for 3 min and 40 cycles of 95 °C/5 s and 60 °C/30 s. Relative quantification was determined by the 2^−ΔΔC^ method [20], using the ribosomal gene 16S as an internal control (primers 534/385).

We specifically measured the relative changes of Firmicutes, Bacteroidetes, Proteobacteria, Actinobacteria, *Prevotella* spp., *Lactobacillus* spp., *Bifidobacterium* spp., *Atopobacter* spp., *Clostridium leptum*, *Clostridium coccoides,* and *Bacteroides fragilis* in each sample. The primer sequences used in the current study are listed in Table 1.

### 2.4. Statistical Analysis

To evaluate the correlation between the gut microbiome profile and clinical indices of each subject, Spearman’s rank correlation coefficient method was used. Mann–Whitney test was used to evaluate significant differences between prostate cancer patients and healthy individuals. Statistical Analyses were performed using Microsoft Excel for Mac V16.60. Values of *p* < 0.05 were considered statistically significant.

## 3. Results

Between November 2019 and March 2021, seven patients scheduled to receive Ra-223 were considered. Of those seven, only four patients met the inclusion criteria. In addition, two samples from one patient were excluded from subsequent analysis due to its low quality. Three patients were included in the final analysis.

Patients and healthy volunteers’ characteristics are summarized in Table 2. Six fecal samples were provided by three prostate cancer patients (age 75 ± 5.86 years) treated with Ra-223, delivered at doses 3894 MBq; 3218 MBq, and 4463 MBq (Table 2).

### 3.1. Gut Microbiota Composition of Prostate Cancer Patients and Healthy Individuals

To compare gut bacterial composition between healthy individuals and prostate cancer patients, we investigated the relative abundance of selected taxa in fecal samples collected from three prostate cancer patients (T0) and from two healthy males.

The relative abundances of the analysed phyla differed between the two groups. Proteobacteria in cancer patients was 11.37-fold higher than in healthy individuals (*p* = 0.035), and Actinobacteria was 1.06-fold higher than that in healthy individuals. In contrast, Bacteroidetes and Firmicutes were 0.76-fold and 0.86-fold lower than those in healthy individuals. At the genus level, *Prevotella, Bifidobacterium,* and *Atopobacter* were increased in prostate cancer, whereas *Lactobacillus* was decreased (0.59-fold). At the species level, *Bacteroides fragilis* was increased (2%), whereas *Clostridium coccoides* (63%) and *Clostridium leptum* (58%) were found to be decreased in prostate cancer patients.

### 3.2. Ra-223 Therapy Impacted the Gut Microbiota of Prostate Cancer Patients

We examined the impact of Ra-223 therapy on prostate cancer patients’ gut microbial community composition. At the phylum level, Firmicutes (11%), Proteobacteria (271%), and Actinobacteria (31%) increased after treatment, whereas Bacteroidetes decreased by 62%.

At the genus level, Atopobacter increased by 124%, whereas Prevotella (70%), Lactobacillus (59%), and Bifidobacterium (69%) decreased after treatment.

At the species level, *Clostridium coccoides* (57%), *Clostridium leptum* (3%) and *Bacteroides fragilis* (90%) decreased after treatment.

### 3.3. The Gut Microbiome of Prostate Cancer Patients Was Not Associated with Clinical Indices

The correlation between each subject’s gut microbiome and clinical indices parameters, including PSA (Prostate-Specific Antigen); ALP (Alkaline phosphatase); LDH (Lactate Dehydrogenase); glucose; calcium; albumin; and urea was analyzed (Table 2). There was no significant correlation between the clinical indices and the gut microbiota composition. In addition, no significant differences were noticed in age, BMI, or treatment dose.

## 4. Discussion

In the current study, we separately compared the relative abundances of selected important taxa of the gut microbiota of healthy individuals with those of prostate cancer patients and the relative abundances of the same taxa before and after Ra-223 treatment in prostate cancer patients.

Previous studies investigated the influence of irradiation from radiotherapy and contaminated areas post-nuclear accidents on the gut microbiota composition [15,16,17,26], however, to the best of our knowledge, there are no published studies regarding the influence of nuclear medicine procedures on the gut microbiota composition.

The use of ionizing radiation to treat multiple diseases is increasing, including nuclear medicine treatments, such as Ra-223.

Our study also showed shifts in the relative abundances of the gut microbiota of prostate cancer patients after the first treatment with Ra-223. The main pathway of elimination is gastrointestinal [10], therefore, changes in the gut microbiota composition were expected.

We found that the most relevant changes were increases in Proteobacteria and Atopobacter. Previous studies with patients treated with pelvic radiotherapy also found that the abundance of Proteobacteria increased after exposure [15,17,26].

Our experiment found decreases of Bacteroidetes, *Prevotella*, *Lactobacillus, Bifidobacterium, Clostridium coccoides, and Bacteroides fragilis*. Previous studies investigating the influence of ionizing radiation found that Bacteroidetes phylum decreased in two studies [15,26] and increased in the other two studies [14,17]. Studies in patients treated with pelvic radiotherapy found that *Bifidobacterium* and *Lactobacillus* were decreased in two studies [27,28] while *Lactobacillus* increased [16]. Nam YD et al. found that *Clostridium leptum* increased after radiotherapy treatments [15] and Wang A et al. found that *Clostridium* cluster XIVa increased after radiotherapy [14], both with significant differences.

The increase of the relative abundance of some taxa can be explained by the fact that some bacteria present more effective intrinsic mechanisms of resistance to radiation, namely their efficient DNA repair mechanisms and their ability to produce protective primary and secondary metabolic products. As an example, Proteobacteria, which was the phyla that increased the most in the present study, have been found to be radioresistant in previous ecological studies in contaminated radioactive areas [29,30].

Previous studies investigated the differences between the gut microbiota of prostate cancer patients and healthy individuals. Che et al. found a higher relative abundance of *Bacteroides massiliensis* and a lower relative abundance of *Faecalibacterium prausnitzii* in prostate cancer patients’ gut microbiota [31]. Golombos et al. 2018 found a higher relative abundance of *Bacteroides massiliensis* and a lower relative abundance of *Feacalibactereium prausnitzii* and *Eubacterium rectale* [7]. Liss et al. and Alanee et al. found enriched Bacteroides spp. in cancer compared with the control group [32]. Liss et al. also found an increased abundance of and *Streptococcus* spp. among patients compared with prostate cancer than in the controls [32].

In the current study, community comparison with phyla-, genera-, and species-level taxa revealed a clear difference between cancer patients and healthy individuals. The most significant differences were the increase of Proteobacteria (by 1063%) and a decrease of *Lactobacillus* (less 41%) *Clostridium leptum* (less 58%), and *Clostridium coccoides* (less 63%).

Proteobacteria is an important phylum composed of Gram-negative bacteria, and commonly associated with dysbiosis. It includes a wide variety of pathogens genera, such as: *Escherichia*, *Salmonella*, *Helicobacter,* and *Legionellales* [33,34]. *Lactobacillus* has been known to be a beneficial bacteria, thus, it has been adopted in the treatment of some gastrointestinal diseases in clinical practice as a probiotic [28,35]. The *Clostridium leptum* (*Clostridium* cluster IV) and *Clostridium coccoides* (from *Clostridium* cluster XIVa) are some of the dominant groups of fecal bacteria in adult humans [36,37], and members of these groups contribute to the production of short chain fatty acids [38].

These results showed an increase of the observed bacteria with known prejudicial effects. Simultaneously, a decrease of the bacteria that are known to be favorable was also seen. These findings are somewhat expected because a change in the health status of the intestinal tract, such as chronic inflammation or abnormal function of the epithelial cells, might directly affect the gut microbial composition [13].

As an example, a recent study found that Proteobacteria was enriched in patients with metastatic prostate cancer and was positively correlated with plasma IL6 level, regional lymph node metastasis status, and distant metastasis status [1].

These differences between the gut microbiota from prostate cancer patients and healthy volunteers might also be due to previous chemotherapy and radiotherapy, which are known to shape intestinal microbiota, causing dysbiosis; and the intestinal microbiota can, in turn, also affect the effectiveness and toxicity of those treatments [10,11].

## 5. Conclusions

In conclusion, the results of this pilot study suggest that Ra-223 could influence the gut microbiota composition. The results confirmed the existing data that the composition of the gut microbiota from prostate cancer patients is different from healthy volunteers, with increased relative abundance of Proteobacteria, known for its pathogenic bacteria, and relevant decreases in bacteria known to be beneficial bacteria.

This study constitutes a pilot study and inherently has limitations, namely, the limited number of participants, which is directly linked to the fact that this treatment option is not frequently used. Our findings should be carefully considered and should be confirmed in larger studies. Due to the innovative character of the findings, we believe this pilot study is relevant, and may pave the way for more robust multicentric studies to unravel the association between Ra233-treated mCRPC and gut microbiota.

## Figures and Tables

**Table 1:** List of primers used for microbiota phylogenetic determination.

Study Ref.	Primers	Target	Sequence	Gram	Phylum	Order
[21]	534/358	16S	ATTACCGCGGCTGCTGG	Bacterial (universal)		
			CCTACGGGAGGCAGCAG			
[22]	Firm	Firmicutes	GGAGYATGTGGTTTAATTCGAAGCA	Gram +	Firmicutes	
			AGCTGACGACAACCATGCAC			
[23]	Lac	*Lactobacillus* spp.	AGCAGTAGGGAATCTTCCA			Lactobacilliales
			CATTYCACCGCTACACATG			
[24]	Atopo	*Atopobacter* spp.	GGGTTGAGAGACCGACC			
			CGGRGCTTCTTCTGCAGG			
[24]	Ccoc	*Clostridium coccoides*	AAATGACGGTACCTGACTAA			Clostridialles
			CTTTGAGTTTCATTCTTGCGAA			
[24]	Clept	*Clostridium leptum*	GCACAAGCAGTGGAGT			
			CTTCCTCCGTTTTGTCAA			
[22]	Act	Actinobacteria	TACGGCCGCAAGGCTA		Actinobacteria	
			TCRTCCCCACCTTCCTCCG			
[23]	Bifid	*Bifidobacterium spp.*	CTCCTGGAAACGGGTGG			
			GGTGTTCTTCCCGATATCTACA			
[22]	Bact	Bacteroidetes	GGARCATGTGGTTTAATTCGATGAT	Gram −	Bacteroidetes	
			AGCTGACGACAACCATGCAG			
[24]	Bfra	*Bacteroides fragilis*	ATAGCCTTTCGAAAGRAAGAT			
			CCAGTATCAACTGCAATTTTA			
[25]	Prevo-F/BacPre-R	*Prevotella* spp.	CRCRCRGTAAACGATGGATG			
			TTGAGTTTCACCGTTGCCGG			
[22]	Prot	Proteobacteria	TCGTCAGCTCGTGTYGTGA		Proteobacteria	
			CGTAAGGGCCATGATG			
[25]	Ent	Enterobacteria	GACCTCGCGAGAGCA			
			CCTACTTCTTTTGCAACCCA			

**Table 2:** Patients and healthy volunteers’ characteristics.

Patient ID	Ra-223 (MBq)	Fecal Sample (mSv/h)	Age (y)	BMI	Number of Drugs	Gleason	Metastases Location	Previous Radiotherapy	Previous Relevant Treatments	Clinical Indices						
										PSA (ng/mL)	ALP (U/L)	LDH (U/L)	Glucose (mg/dL)	Calcium (mEq/L)	Albumin (g/L)	Urea (mg/dL)
**1**	3894	0.41	79	25.51	1	8	Bone	No	Enzalutamide, Docetaxel	160.7	410	415	79	4.8	43.1	45
**2**	3218	0.54	68	20.76	5	9	Bone Lymph nodes	Yes	Abiraterone, Docetaxel	133.5	283	726	132	4.8	39.3	39
**3**	4463	1.21	77	24.76	4	7	Bone	Yes (2001, 2016)	Enzalutamide	2,3	64	127	101	5.1	43.1	45
**C1**			45	29.84	1											
**C2**			38	25.50	0											

BMI—body mass index; PSA–Prostate-specific antigen; ALP—alkaline phosphatase; LDH—Lactate Dehydrogenase. To explore the variation from the before and after treatment samples and from prostate cancer patients and healthy individuals at different taxonomic levels, relative quantification was determined by the 2^−ΔΔCt^ method. We were able to determine the fold-change of the investigated taxa through this method (Table 3).

**Table 3:** The fold-change (FC) of the intervention samples, compared with the unintervention samples, normalized for the reference gene.

	Before/After Ra-223 Treatment					Prostate Cancer Patients/Controls				
Taxa	FC Range (Min–Max)		FC Mean	STDEV	*p*-Value	FC Range (Min–Max)		FC Mean	STDEV	*p*-Value
Firmicutes	0.77	1.64	1.11	0.47	0.729	0.67	1.07	0.86	0.20	0.904
Bacteroidetes	0.12	0.56	0.38	0.23	0.387	0.05	1.42	0.76	0.69	0.674
Proteobacteria	1.92	4.62	3.71	1.55	0.173	4.16	24.72	11.63	11.37	0.035
Actinobacteria	0.48	2.48	1.31	1.04	0.742	0.58	1.55	1.06	0.48	0.641
*Prevotella*	0.01	0.72	0.30	0.37	0.349	0.01	3.69	1.37	2.03	0.779
*Lactobacillus*	0.02	0.89	0.41	0.44	0.664	0.00	1.04	0.59	0.53	0.954
*Bifidobacterium*	0.16	0.39	0.31	0.13	0.311	0.39	1.34	1.01	0.54	0.808
*Atopobacter*	1.03	2.90	2.24	0.95	0.738	1.38	2.09	1.82	0.39	0.132
*Clostridium leptum*	0.71	1.31	0.97	0.31	0.731	0.21	0.55	0.42	0.18	0.354
*Clostridium coccoides*	0.35	0.55	0.43	0.11	0.201	0.13	0.63	0.37	0.25	0.334
*Bacteroides fragilis*	0.01	0.27	0.10	0.14	0.326	0.01	2.98	1.02	1.70	0.834

## Data Availability

Not applicable.
